# Translation and cultural adaptation of the Oxford Shoulder Score to Swedish and validation of a new 7-day recall version in patients with a proximal humerus fracture

**DOI:** 10.2340/17453674.2026.46975

**Published:** 2026-11-12

**Authors:** Per C LUNDIN, Therése F D SUNDH, Lars E ADOLFSSON, Hanna C BJÖRNSSON HALLGREN

**Affiliations:** 1 Department of Orthopaedic Surgery, Linköping University Hospital, Linköping; 2 Department of Biomedical and Clinical Sciences, Linköping University; 3 Department of Physiotherapy, School of Medical Sciences, Faculty of Medicine and Health, Örebro University; 4 Department of Orthopaedic Surgery, Örebro University, Örebro, Sweden

## Abstract

**Background and purpose:**

The Oxford Shoulder Score (OSS) is a widely applied patient-reported outcome measure (PROM) designed to assess function and pain in individuals with shoulder disorders. We primarily aimed to evaluate a translated and culturally adapted Swedish version of the original OSS and secondarily to evaluate a version with a 7-day recall period, intended for rapidly changing conditions such as the early phase following fractures and surgeries.

**Methods:**

The OSS was translated according to Beaton’s guidelines for cross-cultural adaptation. 246 patients with a nonoperatively treated proximal humerus fracture completed the Swedish OSS and the QuickDASH at 1 week (T1, 7-day recall period), 3 months (T2, 7-day recall period), and 12 months (T3, 4-week recall period).

**Results:**

Convergent validity between OSS and QuickDASH (Spearmans’s rho) was rs −0.75 (95% confidence interval [CI] –0.80 to –0.68) at T1, –0.88 (CI –0.91 to –0.84) at T2, and –0.86 (CI –0.90 to –0.81) at T3. Responsiveness for change scores (T2–T1) was –0.77 (CI –0.82 to –0.70) and (T3–T2) –0.80 (CI –0.85 to –0.74). Internal consistency for the respective domains was for pain at T1 0.73 (CI 0.68–0.78), at T2 0.84 (CI 0.80–0.87), at T3 0.87 (CI 0.84–0.89), and for function at T1 0.74 (CI 0.68–0.78), at T2 0.88 (CI 0.86–0.90), at T3 0.91 (CI 0.89–0.92). Intra-rater reliability was ICC = 0.94 (CI 0.90–0.96) between T3 and 2 weeks later.

**Conclusion:**

The Swedish translation of the OSS demonstrated excellent measurement properties. The 7-day recall version demonstrated equivalent good validity, responsiveness, and reliability and seems valuable for monitoring fresh injuries such as proximal humerus fractures.

Patient-reported outcome measures (PROMs) are widely used in orthopedic research and clinical practice [1,2]. To ensure valid use across settings, PROMs require translation, cultural adaptation, and psychometric evaluation, including assessments of validity, reliability, and responsiveness [3-5]. The Oxford Shoulder Score (OSS) is a widely used PROM designed to assess pain and function in shoulder disorders. The OSS was developed by Dawson et al. in 1996, with a 4-week recall period (OSS-4w) [6]. It has since been translated into and validated in several languages, all demonstrating good measurement properties [7-10]. Access to a validated Swedish version would provide a valuable tool for clinicians and researchers, enabling accurate patient-reported data and improved comparability across studies and healthcare settings. Lillkrona and Salomonsson translated OSS into Swedish 2004, but no validation was published. As it is important to establish psychometric properties and cross-cultural equivalence, a new translation was developed and validated.

PROM results can be influenced by recall period; changes in symptoms or function during the recall period may introduce recall bias and affect accuracy of the reported outcomes. For rapidly changing conditions, a recall period shorter than 4 weeks may be preferable [11], as previously demonstrated by the evaluation of a 7-day recall version of the Oxford Elbow Score intended for acute elbow injuries [12]. The same adjustment of OSS to a 7-day recall period was performed to capture changes in the early stages after a shoulder injury. The aim was primarily to investigate the properties of a translated and culturally adapted Swedish version of OSS and secondarily to investigate the properties of a shortened 7-day recall period (OSS-7d).

## Methods

### Study design, setting, and eligibility criteria for validation process

This study was based on the first 350 patients enrolled in the randomized controlled NOSWEPH (Non-Operative treatment of SWEdish Proximal Humerus fractures) trial with data collected from 4 hospitals in Sweden: Linköping University Hospital, Kalmar Hospital, Västervik Hospital, and Örebro University Hospital between February 2019 and October 2023 (NCT03786679). The inclusion criterion was a PHF treated nonoperatively within 10 days after trauma. The cohort included all types of fractures. According to Neer classification, 137 with displacement < 1 cm or angulation/rotation less than 45°, 53 x 2-part, 40 x 3-part, and 16 x 4-part fractures. All patients provided written and informed consent to participate in the study. The exclusion criterion was incompletely filled out OSS and/or QuickDASH questionnaires at baseline, 3-, and 12-months’ follow-up. Responses with 1 or more missing items were excluded [13].

The study is reported in accordance with the COSMIN guidelines.

### Translation and adaptation of the OSS

To establish psychometric properties and cross-cultural equivalence, a new Swedish translation was developed independently of the previous version. Permission to use and translate the OSS was obtained from Oxford University Innovation Ltd, and the translation followed the guidelines proposed by Beaton et al. [14]. 2 independent forward translations into Swedish were performed by native Swedish speakers fluent in English and synthesized into a single version. This version was then back-translated by 2 native English speakers fluent in Swedish. The back translation was compared with the original questionnaire by an independent translator, and any discrepancies were resolved by consensus among the translators and authors to ensure linguistic and conceptual equivalence. A local committee of bilingual experts within the field of medicine reviewed the process and comprehensiveness of the prefinal translated version, which was then tested on a pilot group of patients with proximal humeral fracture (PHF) with no need for further changes. Oxford University Innovation Ltd was informed and the validation process approved.

### Data collection

246 patients were included, 219 (89%) women and 27 (11%) men, with median age 72 years (range 26–95). At the orthopedic clinic the questionnaires were completed on a laptop without the assistance of the attending physician and recorded in the online database WebCrf provided by the University of Trondheim. Patients responded to OSS and QuickDASH at 3 different time points: post injury within 10 days (T1, 7-day recall), at 3 months (T2, 7-day recall), and at 12 months (T3, 4-weeks’ recall). To evaluate a 7-day recall period in OSS the modified question “During the past week” was used at T1 and T2 and the original question “During the past 4 weeks” at T3. Intra-rater reliability was assessed by randomly selecting 81 of the 246 included patients, who completed OSS twice in a stable phase: first at T3, and then 2 weeks later, provided by regular mail in a pre-paid envelope.

### Outcome measures

The OSS consists of 12 items in 2 domains, including 4 items grading pain severity and 8 items with a range of shoulder-specific activities. Each item is scored on a 5-point Likert scale. Total scoring is 0–48; a higher score indicates better shoulder function and less pain [15].

The Quick Disabilities of the Arm, Shoulder and Hand (QuickDASH) is an 11-item PROM designed to evaluate physical function, pain, and psychosocial properties. Each item is rated on a 5-point Likert scale, and the total score is transformed to a 0–100 scale in which higher scores indicate greater disability and symptom severity [16].

### Statistics

Statistical analyses were conducted using IBM SPSS Statistics for Windows, Version 27 (IBM Corp, Armonk, NY, USA). Statistical significance was defined as a P value < 0.05. Measurement properties were evaluated in accordance with the COSMIN checklist and terminology [3]. The present study employed QuickDASH, but comparisons with studies utilizing DASH and Constant–Murley Score was considered justified, as those instruments have proven strong concurrent validity [17,18]. Convergent validity between OSS and Quick DASH at T1, T2, and T3 was investigated by using Spearman’s correlation coefficients (Spearman’s rho, rs). As lower QuickDASH scores indicate better outcomes whereas higher OSS scores indicate better outcomes, correlations were expected to yield negative values. Correlations were arbitrarily interpreted as negligible (r < –0.3), low (–0.3 ≤ r < –0.5), moderate (–0.5 ≤ r < –0.7), high (–0.7 ≤ r < –0.9), or very high (–0.9 ≤ r ≤ –1.0) according to the recommendations by Mukaka [19]. Based on prior research, the predefined hypothesis was that correlations between QuickDASH and OSS scores would be at least r ≥ 0.75 to support convergent validity [20]. To evaluate structural validity, 2 a priori models were formulated: a unidimensional model including all 12 OSS items loading on a single latent factor to assess whether the Swedish OSS retained its intended unidimensional structure, and a 2-factor model consisting of Pain (items 1, 8, 11, and 12) and Function (items 2–7, 9, and 10) to explore whether pain- and function-related items represented distinguishable but related dimensions, consistent with the approach used by Dawson et al. [21]. Confirmatory factor analyses were conducted using R (version 4.5.2; R Core Team, 2025; R Foundation for Statistical Computing, Vienna Austria) with the lavaan package. Model fit was investigated using the Comparative Fit Index (CFI), Tucker–Lewis Index (TLI), root mean square error of approximation (RMSEA), and standardized root mean square residual (SRMR). Good model fit was defined according to commonly recommended thresholds: CFI and TLI ≥ 0.95, RMSEA < 0.06, and SRMR < 0.08. OSS items were considered as ordered categorical variables. Analyses were based on estimated item thresholds and polychoric correlations [22]. Responsiveness, defined as longitudinal validity [21], was examined by calculating change scores between time points: T1 and T2 (T2 minus T1), T2 and T3 (T3 minus T2). Spearman’s rho was used to assess correlations between change scores of the OSS and Quick DASH for both intervals. Effect size (ES) and standardized response mean (SRM) were calculated to further evaluate responsiveness. Based on previous research by Jonsson et al. [12] a general hypothesis of r ≥ 0.60 was formulated for correlations between change scores. Patients were expected to improve over time. Accordingly, OSS scores were expected to increase, whereas QuickDASH scores were expected to decrease. Therefore, a negative correlation between the respective change scores was hypothesized. Internal consistency of the OSS domains at T1, T2, and T3 was assessed using Cronbach’s alpha coefficient, and based on previous recommendation an acceptable threshold of > 0.7 was set [23]. Intra-rater reliability was assessed with an intraclass correlation coefficient (ICC). Based on the 95% confidence interval (CI) of the ICC estimate, values less than 0.5, between 0.5 and 0.75, between 0.75 and 0.9, and greater than 0.90 are indicative of poor, moderate, good, and excellent reliability, respectively [24]. ICC ≥ 0.70 was interpreted as acceptable. Inter-rater reliability was assessed using a two-way mixed-effects model based on a consistency definition, under Shrout and Fleiss’s convention. Floor and ceiling effects were considered present if more than 15% of respondents achieved the lowest or highest possible score, in accordance with established criteria [25].

### Ethics, funding, and disclosures

The study was approved by the Central Ethical Review Board, Stockholm 2018-12-19 (Dnr 2021-01328). The authors report no conflict of interest. The NOSWEPH study was funded by the Medical Research Council of Southeast Sweden (FORSS- 06080198) and HCBH was funded by ALF Grants Region Östergötland (RÖ-999163). TFDS was funded by ALF Grants Region Örebro (OLL-998923). LEA was partially supported by Linköping and Örebro Universities. There are no financial interests or other relationships that may lead to a conflict of interest concerning this study. No important aspects of this study have been omitted. All authors had full access to all the data and data analysis. Complete disclosure of interest forms according to ICMJE are available on the article page, doi: 10.2340/17453674.2026.46975

## Results

### Total scores

Mean scores were for OSS and QuickDASH respectively at T1, OSS 9 (CI 8.1–9.6), QuickDASH 79 (CI 77.4–80.6), at T2 OSS 36 (CI 35.0–37.2), QuickDASH 28 (CI 25.7–30.4), and at T3 OSS 41 (CI 40.1–42.1), QuickDASH 16 (CI 13.4-17.7) (Table 1).

**Table 1:** Results of the OSS and QuickDASH at T1, T2, and T3

Item	T1 ^a^ mean (SD) [range]	Difference (T2–T1) CI ^d^	T2 ^b^ mean (SD) [range]	Difference (T3–T2) CI ^d^	T3 ^c^ mean (SD) [range]
OSS					
Total score	8.9 (6.0) [0–40]	26–28	36 (8.6) [6–48]	4.1–6.0	41 (8.0) [7–48]
Pain	3.2 (2.7) [0–16]	6.8–7.7	11 (3.5) [2–16]	1.7–2.6	12 (3.5) [2–16]
Shoulder function	5.7 (4.0) [0–27]	19–21	26 (5.8) [2–32]	2.2–3.5	29 (5.2) [2–32]
QuickDASH	79 (13) [9–100]	49–59 **^e^**	28 (19) [0–86]	10–15 **^e^**	16 (17) [0–82]

CI = 95% confidence interval; DASH = Disabilities of the Arm, Shoulder and Hand; OSS = Oxford Shoulder Score; SD = standard deviation.

a T1, < 10 days after injury.

b T2, 3 months after injury.

c T3, 1 year after injury.

d T-test.

e Reverse difference (T1–T2) and (T2–T3).

### Construct validity

Convergent validity, the correlation between OSS and QuickDASH, was for the total score Spearman’s rho rs −0.75 (CI –0.80 to –0.68) at T1, –0.88 (CI –0.91 to –0.84) at T2, and –0.86 (CI –0.90 to –81) at T3. For the different domains, OSS pain vs QuickDASH was rs −0.73 (CI –0.78 to –0.66) at T1, –0.77 (CI –0.82 to –0.70) at T2, and –0.76 (CI –0.82 to –0.70) at T3 and OSS function vs QuickDASH was rs −0.62 (CI –0.82 to –0.70) at T1, -0.84 (CI –0.88 to –0.79) at T2, and –0.80 (CI –0.85 to –0.74) at T3 ranging from moderate (–0.5 ≤ r < –0.7) to high (–0.7 ≤ r < –0.9) correlation (Table 2).

**Table 2:** Assessment of convergent validity based on data at T1, T2, and T3 with Spearman’s rank correlation coefficients (rho) between the OSS (its domains) and QuickDash

Item	OSS – Total score rho (CI)	OSS – Pain rho (CI)	OSS – Function rho (CI)
T1 (1-week recall)			
OSS – Pain	0.83 (0.78 to 0.87)	–	–
OSS – Function	0.92 (0.88 to 0.94)	–	–
QuickDASH	–0.75 (–0.80 to –0.68)	–0.73 (–0.78 to –0.66)	–0.62 (–0.70 to –0.53)
T2 (1-week recall)			
OSS – Pain	0.88 (0.85 to 0.91)	–	–
OSS – Function	0.94 (0.92 to 0.95)	–	–
QuickDASH	–0.88 (–0.91 to –0.84)	–0.77 (–0.82 to –0.70)	–0.84 (–0.88 to –0.79)
T3 (4-week recall)			
OSS – Pain	0.92 (0.90 to 0.94)	–	–
OSS – Function	0.87 (0.81 to 0.91)	–	–
QuickDASH	–0.86 (–0.90 to –0.81)	–0.76 (–0.82 to –0.70)	–0.80 (–0.85 to –0.74)

For abbreviations, see Table 1.

All correlation is significant at the 0.01 level (2-tailed).

We used bootstrapping to calculate confidence intervals based on 1,000 bootstrap samples.

Structural validity investigated using CFA; at T1 (OSS-7d), the 1-factor model demonstrated acceptable fit (CFI 0.968, TLI 0.961, RMSEA 0.073, SRMR 0.090) and the 2-factor model showed good fit across all indices (CFI 0.984, TLI 0.980, RMSEA 0.052, SRMR 0.072). At T1 (OSS-7d) the 2-factor model showed significantly better fit than the 1-factor model (P < 0.001). At T3 follow-up (OSS-4 w), both models demonstrated good to excellent fit, but significantly (P < 0.001) superior fit for the 2-factor model (CFI 0.999, TLI 0.999, RMSEA 0.027, SRMR 0.042) compared with the 1-factor model (CFI 0.995, TLI 0.994, RMSEA 0.075, SRMR 0.064). Factor loadings were generally moderate to high across both assessments. The 1-factor model standardized loadings at T1 (OSS-7d) ranged from 0.449 to 759, and at T3 (OSS-4 week) from 0.637 to 0.935. For Function and Pain domains, standardized loading for the 2-factor model at T1 (OSS-7d) ranged from 0.527 to 0.822, and at T3 (OSS-4 week), ranged from 0.665 to 0.953 (for factor loading Tables, see Supplementary data). The Pain and Function factors were strongly correlated at both timepoints (T1 r = 0.77, CI 0.70–0.84; T3 r = 0.87, CI 0.84–0.91), indicating substantial overlap between the constructs.

### Responsiveness

Responsiveness was assessed by examining correlations between change scores of the Swedish OSS and QuickDASH. A priori, a correlation coefficient of at least 0.60 was considered indicative of adequate responsiveness. The observed correlations exceeded this predefined threshold, supporting that the Swedish OSS is responsive to clinically relevant changes over time. Spearman’s rho for total change scores from T1 to T2 (T2–T1) was –0.77 (CI –0.82 to –0.70) and from T2 to T3 (T3–T2) –0.8 (CI –0.85 to –0.74). For domains, change score OSS pain vs QuickDASH from T1 to T2 (T2–T1) was –0.65 (CI –0.72 to –0.56) and from T2 to T3 (T3–T2) –0.70 (CI –0.62 to –0.73). Change score OSS function vs QuickDASH from T1 to T2 (T2–T1) was –0.74 (CI –0.79 to –0.66) and from T2 to T3 (T3–T2) –0.73 (CI –0.79 to –0.65) (Table 3). Effect size (ES) at T1 to T2 was for the total OSS 4.58, for pain 2.69, and for function 4.95. For T2 to T3 the ES for the total OSS was 0.84, for pain 0.80, and for function 0.70. Standardized response mean (SRM) at T1 to T2 was for the total OSS 3.08, for pain 2.02, and for function 3.13. For T2 to T3 the ES for the total OSS was 0.66, for pain 0.63, and for function 0.57.

**Table 3:** Responsiveness (longitudinal validity) assessed with Spearman’s correlation coefficients (rho) between T1/T2 and T2/T3 change scores of the OSS and QuickDash

Change scores	OSS – Total score rho (CI)	OSS – Pain rho (CI)	OSS – Function rho (CI)
T1/T2			
OSS – Pain	0.86 (0.81 to 0.89)	–	–
OSS – Function	0.94 (0.92 to 0.96)	0.65 (0.57 to 0.72)	–
QuickDASH	–0.77 (–0.82 to –0.70)	–0.65 (–0.72 to –0.56)	–0.74 (–0.79 to –0.66)
T2/T3			
OSS – Pain	0.86 (0.82 to 0.89)	–	–
OSS – Function	0.91 (0.88 to 0.94)	0.60 (0.50 to 0.68)	–
QuickDASH	–0.80 (–0.85 to –0.74)	–0.70 (–0.62 to –0.73)	–0.73 (–0.79 to –0.65)

For abbreviations, see Table 1.

### Internal consistency

Internal consistency was evaluated using Cronbach’s α, with a predefined threshold of ≥ 0.70 considered acceptable. The observed values exceeded this threshold, indicating adequate internal consistency of the Swedish OSS. Cronbach’s α for the entire OSS (pain and function domains) was at T1 0.81 (CI 0.77–0.84), at T2 0.91 (CI 0.89–0.93), and at T3 0.92 (CI 0.91–0.94). For the pain domain, Cronbach’s α was at T1 0.73 (CI 0.68–0.78), at T2 0.84 (CI 0.80–0.87), at T3 0.87 (CI 0.84–0.89), and for domain function at T1 0.74 (CI 0.68–0.78), at T2 0.88 (CI 0.86–0.90), and at T3 0.91 (CI 0.89–0.92). Removal of individual items did not markedly improve Cronbach’s α, suggesting that all items contributed to the total score.

### Intra-rater reliability

ICC is interpreted as acceptable when at least 0.70. The ICC for the OSS total score was 0.94 (CI 0.90–0.96) P < 0.001.

### Floor and ceiling effects

Floor and ceiling effects were low at T1 with 1.2% and 0.4%, and at T2 with 0% and 4.9% of the participants achieving the lowest and highest scores respectively. At T3, 0% had the lowest score and 22% had maximum score.

## Discussion

We present a Swedish translation of the OSS and evaluation of measurement properties using both the original 4-week recall period (OSS-4w) and a shortened 7-day version (OSS-7d) intended for fresh injuries and conditions with rapid changes. The primary finding was that the OSS-4w demonstrated excellent measurement properties, and the OSS-7d similarly strong performance. Convergent validity was supported by strong correlations with QuickDASH, meeting the a priori hypothesis at T1 (–0.75), surpassed at T2 (–0.88) and at T3 (–0.86). Correlations between QuickDASH and the 2 OSS domains, pain and shoulder function, was acceptable at all time points. Compared with previous studies validating the OSS-4w using DASH or QuickDASH, observed correlations were comparable, both with OSS-4w and OSS-7d. OSS is often conceptualized as measuring both pain and function, but previous research has suggested a predominantly unidimensional structure [7,26]. Correlations between OSS domains should be interpreted cautiously, as results regarding its structural validity vary and a strong domain correlation likely reflects a close relationship between pain and function rather than a distinct underlying construct [7]. The CFA findings in our study support the structural validity of the Swedish OSS and indicate that both a unidimensional and a correlated 2-factor structure provide acceptable fit. However, the 2-factor model consistently demonstrated superior fit across all indices at both follow-up timepoints suggesting that separating pain and function may better reflect the underlying structure of the instrument. Despite this, the strong correlations between pain and function indicate substantial conceptual overlap between domains, particularly at the T3 assessment. These findings suggest that the OSS primarily behaves as a unidimensional measure for overall shoulder-related disability, while still allowing identification of clinically relevant pain and function. This interpretation is consistent with previous validation studies of the OSS, where high inter-factor correlations and strong internal consistency have supported use of the total score despite evidence of multidimensionality [21,26]. All findings from ourstudy are consistent with previous OSS validation studies. The Korean and Chinese adaptations using DASH also demonstrated high internal consistency, good test–retest reliability, and strong correlations with related measures, supporting convergent validity. Similarly, the Polish validation reported excellent reliability and strong correlations with QuickDASH, further confirming robustness of the translated instrument [7,8,10].

Responsiveness demonstrated favorable measurement characteristics, with correlation coefficients exceeding the a priori threshold of ≥ 0.6 for both the OSS total score and its subdomains. ES and SRM indicated very strong responsiveness at T1–T2 and moderate to strong responsiveness at T2–T3, supporting that the Swedish OSS is sensitive to clinical change following PHFs, especially during the first 3 months. OSS-4w and OSS 7-d also demonstrated good reliability with Cronbach’s alpha values for the OSS domains falling within the recommended range of 0.70–0.95 [25], indicating satisfactory internal cohesion of the instrument. Intra-rater reliability, assessed using intraclass correlation coefficients, was excellent, as repeated administration of the instrument produced highly consistent results (ICC > 0.9) [24]. The 2-week interval following the 12-month follow-up was selected to minimize the risk of changes in clinical status while allowing sufficient time to reduce recall of the initial responses [3].

The choice of recall period is critical in patient-reported outcome measurement and should reflect both characteristics of the condition and the purpose of assessment. Short recall periods may underestimate symptom burden in fluctuating conditions, whereas longer periods increase the risk of recall bias. The optimal recall period depends on symptom variability, study design, intended use of the instrument, and the nature of the condition. Consequently, the same instrument may require different recall periods at different time points, and recall period selection should be guided by both clinical and methodological considerations [12,27,28]. Coles et al. observed that nearly one-third of respondents extend their recall period beyond 7 days even when instructed otherwise, which emphasizes the need for clearly defined recall periods to minimize bias [29]. The original OSS uses a 4-week recall period, developed for chronic or stable shoulder conditions. In acute injuries and early recovery when symptoms may change rapidly, a shorter recall period may better reflect current status and reduce recall bias. The Swedish OSS was administered with a 7-day recall at T1 and T2, and with the original 4-week recall at T3. The strong correlations between OSS and QuickDASH change scores suggest that both recall periods captured meaningful clinical change. The inclusion of a shortened 7-day recall version provides a useful complement to the original instrument, particularly for documenting pre- and post-injury status. However, scores obtained using different recall periods may not be directly interchangeable, and the 7-day version could primarily be regarded as a complementary measure rather than a replacement of the standard instrument. While the shortened recall may be advantageous in acute or early follow-up settings, its applicability to long-term outcome assessment may be limited. Peasgood et al. found that longer recall periods were generally associated with higher symptom severity and poorer Health-Related Quality-of-Life scores, while Masud et al. demonstrated that recall accuracy declines over time and becomes unreliable beyond 3 months postoperatively. All these findings highlight the importance of selecting an appropriate recall period to minimize recall bias and improve measurement precision [30,31].

A ceiling effect was observed at 1-year follow-up, with 22% of participants achieving the maximum OSS score. This exceeds the commonly accepted 15% threshold and suggests reduced sensitivity of the instrument in distinguishing between patients with high levels of recovery, but this is not unexpected in populations with substantial improvement over time. The number of patients included in the present study is larger than in most similar studies, reinforcing the findings [7,8,10].

### Limitations

A notable limitation of our study is the predominance of female participants, which may affect the generalizability. Furthermore, since the study sample consisted of older individuals with proximal humeral fractures, this may potentially influence comparability with younger populations or patients with other shoulder pathologies.

### Conclusions

The Swedish translation of the OSS demonstrated excellent measurement properties, with strong validity, responsiveness, and reliability when used with the original 4-week recall period (OSS-4w). The 7-day recall version (OSS-7d) performed comparably to the original 4-week recall period and may be particularly valuable for monitoring fresh injuries, while the 4-week recall remains appropriate for long-term follow-up.

Future studies should establish minimal important change (MIC) values and examine responsiveness across broader diagnostic categories.

### Supplementary data

Factor loading Tables are available as supplementary data on the article home page, doi: 10.2340/17453674.2026.46975

## Supplementary Material



## Notes

PCL: writing—review and editing, design of the study, data collection, data analysis, interpretation of the results. TFDS: writing—review and editing, data collection, interpretation of the results. LEA: conceptualization and design of the study, interpretation of the results, critical revisions of the manuscript, and funding acquisition. HCBH: conceptualization and design of the study, interpretation of the results, critical revisions of the manuscript, and funding acquisition.

The authors would like to thank Terez Zara Hanqvist, research nurse at the Department of Orthopaedics, Linköping University Hospital, for administration work.

Handling co-editors: Jeppe Vejlgaard Rasmussen and Robin Christensen

*Acta* thanks Agnieszka Bejer and Niko Kämppä for help with peer review of this manuscript.

## References

[1] Brorson S, Borg S A, Houkjær L L, Holtz K B, Issa Z. Patient-reported outcome was close to the Danish background population 6 months after non-surgical treatment of Neer 2-part surgical neck fractures: a prospective cohort study in patients aged 60 or above. Acta Orthop 2024; 95: 619-24. doi:10.2340/17453674.2024.42301. PMC11538501 39499244

[2] Südkamp N, Jaeger M, Adolfsson L, Berndt T, Blauth M, Joeris A, et al. Total shoulder arthroplasty for glenohumeral osteoarthritis leads to better outcomes than hemiarthroplasty at a minimum 5 years: an intraoperative randomization-controlled trial of 79 patients. Acta Orthop 2025; 96: 904-13. doi:10.2340/17453674.2025.44946. PMC12697190 41379463

[3] Mokkink L B, Terwee C B, Patrick D L, Alonso J, Stratford P W, Knol D L, et al. The COSMIN study reached international consensus on taxonomy, terminology, and definitions of measurement properties for health-related patient-reported outcomes. J Clin Epidemiol 2010; 63: 737-45. doi: 10.1016/j.jclinepi.2010.02.006. 20494804

[4] Lowry K J, Brox W T, Naas P L, Tubb C C, Muschler G F, Dunn W. Musculoskeletal-based patient-reported outcome performance measures, where have we been—where are we going. J Am Acad Orthop Surg 2019; 27: e589-e595. doi: 10.5435/JAAOS-D-18-00429. 31232793

[5] Lee M K, Zaniletti I, Larson D R, Lewallen D G, Berry D J, Maradit Kremers H. Nuts and bolts of patient-reported outcomes in orthopaedics. J Arthroplasty 2023; 38: 616-21. doi: 10.1016/j.arth.2022.11.011. PMC10010940 36481287

[6] Dawson J, Fitzpatrick R, Carr A. Questionnaire on the perceptions of patients about shoulder surgery. J Bone Joint Surg Br 1996; 78: 593-600. PMID: 8682827.

[7] Xu X, Wang F, Wang X, Wei X, Wang Z. Chinese cross-cultural adaptation and validation of the Oxford shoulder score. Health Qual Life Outcomes 2015; 13: 193. doi: 10.1186/s12955-015-0383-5. PMC4668609 26631074

[8] Bejer A, Szczepanik M, Płocki J, Szymczyk D, Kulczyk M, Pop T. Translation, cross-cultural adaptation and validation of the polish version of the Oxford Shoulder Score in patients undergoing arthroscopic rotator cuff repair. Health Qual Life Outcomes 2019; 17: 191. doi: 10.1186/s12955-019-1263-1. PMC6937923 31888693

[9] Fang Y Y, Ackerman I N, Page R, Harris I A, Cashman K, Lorimer M, et al. Measurement properties of the Oxford shoulder score and minimal clinically important changes after primary total shoulder replacement surgery. Arthritis Care Res (Hoboken) 2024; 76: 895-903. doi: 10.1002/acr.25304. 38258339

[10] Roh Y H, Noh J H, Kim W, Oh J H, Gong H S, Baek G H. Cross-cultural adaptation and validation of the Korean version of the Oxford shoulder score. Arch Orthop Trauma Surg 2012; 132: 93-9. doi: 10.1007/s00402-011-1393-3. 21959695

[11] Schneider S, Broderick J E, Junghaenel D U, Schwartz J E, Stone A A. Temporal trends in symptom experience predict the accuracy of recall PROs. J Psychosom Res 2013; 75: 160-6. doi: 10.1016/j.jpsychores.2013.06.006. PMC3740272 23915773

[12] Jonsson E, Wänström J, Björnsson Hallgren H, Adolfsson L. The Oxford Elbow Score demonstrated good measurement properties when used with a shortened 7-day recall period. JSES Int 2023; 7: 499-505. doi: 10.1016/j.jseint.2022.12.023. PMC10229418 37266162

[13] Beaton D E, Wright J G, Katz J N. Development of the QuickDASH: comparison of three item-reduction approaches. J Bone Joint Surg Am 2005; 87: 1038-46. doi: 10.2106/jbjs.D.02060. 15866967

[14] Beaton D E, Bombardier C, Guillemin F, Ferraz M B. Guidelines for the process of cross-cultural adaptation of self-report measures. Spine 2000; 25: 3186-91. doi: 10.1097/00007632-200012150-00014. 11124735

[15] Dawson J, Rogers K, Fitzpatrick R, Carr A. The Oxford shoulder score revisited. Arch Orthop Trauma Surg 2009; 129: 119-23. doi: 10.1007/s00402-007-0549-7. 18183410

[16] Gummesson C, Ward M M, Atroshi I. The shortened disabilities of the arm, shoulder and hand questionnaire (QuickDASH): validity and reliability based on responses within the full-length DASH. BMC Musculoskelet Disord 2006; 7: 44. doi: 10.1186/1471-2474-7-44. PMC1513569 16709254

[17] Pyörny J, Sletten I N, Jokihaara J. Concurrent validity study of QuickDASH with respect to DASH in patients with traumatic upper extremity amputation. BMC Musculoskelet Disord 2024; 25: 86. doi: 10.1186/s12891-024-07183-w. PMC10804815 38263085

[18] Fayad F, Lefevre-Colau M M, Gautheron V, Macé Y, Fermanian J, Mayoux-Benhamou A, et al. Reliability, validity and responsiveness of the French version of the questionnaire Quick Disability of the Arm, Shoulder and Hand in shoulder disorders. Man Ther 2009; 14: 206-12. doi: 10.1016/j.math.2008.01.013. 18436467

[19] Mukaka M M. Statistics corner: a guide to appropriate use of correlation coefficient in medical research. Malawi Med J 2012; 24: 69-71. PMID: 23638278. PMC3576830

[20] Kruger N, Stander L, Maqungo S, Roche S, Held M. The Oxford Shoulder Score: cross-cultural adaptation and translational validation into Afrikaans. SA Orthop J 2018; 17. doi: 10.17159/2309-8309/2018/v17n1a2.

[21] Dawson J, Harris K K, Doll H, Fitzpatrick R, Carr A. A comparison of the Oxford shoulder score and shoulder pain and disability index: factor structure in the context of a large randomized controlled trial. Patient Relat Outcome Meas 2016; 7: 195-203. doi: 10.2147/prom.S115488. PMC5123655 27920590

[22] Flora D B, Curran P J. An empirical evaluation of alternative methods of estimation for confirmatory factor analysis with ordinal data. Psychol Methods 2004; 9: 466-91. doi: 10.1037/1082-989x.9.4.466. PMC3153362 15598100

[23] Cronbach L J. Coefficient alpha and the internal structure of tests. Psychometrika 1951; 16: 297-334. doi: 10.1007/bf02310555.

[24] Koo T K, Li M Y. A Guideline of Selecting and reporting intraclass correlation coefficients for reliability research. J Chiropr Med 2016; 15: 155-63. doi: 10.1016/j.jcm.2016.02.012. PMC4913118 27330520

[25] Terwee C B, Bot S D, de Boer M R, van der Windt D A, Knol D L, Dekker J, et al. Quality criteria were proposed for measurement properties of health status questionnaires. J Clin Epidemiol 2007; 60: 34-42. doi: 10.1016/j.jclinepi.2006.03.012. 17161752

[26] Simpson J, Keding A, Spencer S, Brealey S, Rangan A. Factor structure of the Oxford Shoulder Score: secondary analyses of the UK FROST and PROFHER trial populations. J Orthop Surg Res 2023; 18: 846. doi: 10.1186/s13018-023-04319-x. PMC10631035 37940977

[27] Food and Drug Administration. Guidance for industry: Patient-reported outcome measures: use in medical product development of support labeling claims. Federal Register; 2009. Available from: https://www.fda.gov/regulatory-information/search-fda-guidance-documents/patient-reported-outcome-measures-use-medical-product-development-support-labeling-claims 10.1186/1477-7525-4-79 PMC1629006 17034633

[28] Norquist J M, Girman C, Fehnel S, DeMuro-Mercon C, Santanello N. Choice of recall period for patient-reported outcome (PRO) measures: criteria for consideration. Qual Life Res 2012; 21: 1013-20. doi: 10.1007/s11136-011-0003-8. 21909804

[29] Coles T, Plyler K, Hernandez A, Fillipo R, Henke D M, Arizmendi C, et al. Recalling what we thought we knew about recall periods: a qualitative descriptive study of how adults diagnosed with cancer use recall periods for patient-reported outcome items about physical function. Qual Life Res 2025; 34: 857-66. doi: 10.1007/s11136-024-03847-1. PMC11919987 39630392

[30] Peasgood T, Caruana J M, Mukuria C. Systematic review of the effect of a one-day versus seven-day recall duration on patient reported outcome measures (PROMs). Patient 2023; 16: 201-21. doi: 10.1007/s40271-022-00611-w. PMC10121527 36786931

[31] Masud S, Piche J D, Muralidharan A, Nassr A, Aleem I. Do patients accurately recall their preoperative symptoms after elective orthopedic procedures? Cureus 2023; 15: e36810. doi: 10.7759/cureus.36810. PMC10135438 37123705

