# Supplementary material for: Translation and cultural adaptation of the Oxford Shoulder Score to Swedish and validation of a new 7-day recall version in patients with a proximal humerus fracture
Source: Acta Orthop. 2026 Nov 12;97:799–805. doi: 10.2340/17453674.2026.46975 (PMC13756492; doi:10.2340/17453674.2026.46975)
Supplement: Supplementary file 1 [file ActaO-97-46975-s1.pdf]

Supplementary data

Factor loading for 1 and 2-factor models, OSS-7d and OSS-4 week

Factor loading 1-factor model (OSS-7d)

Sr no.	Factor	Item	Loading
1	OSS	OSS 1	0.651
2	OSS	OSS 2	0.692
3	OSS	OSS 3	0.565
4	OSS	OSS 4	0.679
5	OSS	OSS 5	0.630
6	OSS	OSS 6	0.533
7	OSS	OSS 7	0.499
8	OSS	OSS 8	0.697
9	OSS	OSS 9	0.565
10	OSS	OSS 10	0.654
11	OSS	OSS 11	0.759
12	OSS	OSS 12	0.591

Factor loading 1-factor model (OSS-4 week)

Sr no.	Factor	Item	Loading
1	OSS	OSS 1	0.935
2	OSS	OSS 2	0.838
3	OSS	OSS 3	0.841
4	OSS	OSS 4	0.807
5	OSS	OSS 5	0.758
6	OSS	OSS 6	0.837
7	OSS	OSS 7	0.838
8	OSS	OSS 8	0.880
9	OSS	OSS 9	0.875
10	OSS	OSS 10	0.816
11	OSS	OSS 11	0.898
12	OSS	OSS 12	0.637

Factor loading 2-factor model (OSS-7d)

Sr no.	Factor	Item	Loading
1	Pain	OSS 1	0.695
2	Pain	OSS 8	0.739
3	Pain	OSS 11	0.822
4	Pain	OSS 12	0.639
5	Function	OSS 2	0.738
6	Function	OSS 3	0.594
7	Function	OSS 4	0.709
8	Function	OSS 5	0.657
9	Function	OSS 6	0.561
10	Function	OSS 7	0.527
11	Function	OSS 9	0.590
12	Function	OSS 10	0.686

Factor loading 2-factor model (OSS-4 week)

Sr no.	Factor	Item	Loading
1	Pain	OSS 1	0.953
2	Pain	OSS 8	0.894
3	Pain	OSS 11	0.938
4	Pain	OSS 12	0.665
5	Function	OSS 2	0.861
6	Function	OSS 3	0.867
7	Function	OSS 4	0.827
8	Function	OSS 5	0.777
9	Function	OSS 6	0.857
10	Function	OSS 7	0.853
11	Function	OSS 9	0.895
12	Function	OSS 10	0.833
